# County Hospital Surgical Clinic

**Published:** 1866-11

**Authors:** Geo. K. Amerman

**Affiliations:** Attending Surgeon


### HOSPITAL REPORTS.

### COUNTY HOSPITAL SURGICAL CLINIC.

*Strictures of the Urethra—Perineal Section.* By GEO. K. AMERMAN, M. D., Attending Surgeon.

GENTLEMEN,—To-day, we shall occupy the greater portion of the hour in studying the causes, symptoms and treatment of strictures of the urethra. This affection, though not of very frequent occurrence, is still sufficiently so to demand your careful consideration; and if you would make yourselves competent to manage a bad case, you must first thoroughly understand the subject, and possess, in addition, patience, perseverance and skill.

In the study of this subject, and to make it as simple as possible, let me direct your attention to the patient now before you. He is 53 years old, of moderately temperate habits, and has always enjoyed good health until about three years ago. At that time, he began to experience difficulty in passing water. It would often come in a small stream, followed by a sense of scalding, with an uneasiness about the loins and perineum. After exposure or much exercise, these symptoms were greatly aggravated, so that he was very soon induced to consult a surgeon, whose treatment afforded so much relief, that he supposed he was entirely cured. Last September, about eight months ago, he had an attack of intermittent fever, and was obliged to undergo severe hardship and exposure, which brought on a relapse of the old difficulty with more severity than ever, and after exhausting all his means to obtain relief, he was finally obliged to ask admission into the Hospital.

He is now unable to pass his water, and has very little control of the urinary organs. The urine is constantly dribbling away from him, (incontinence,) and he suffers great pain in the loins, penis and perineum.

These symptoms are sufficiently characteristic to enable us to form a correct diagnosis, nevertheless, they cannot be regarded as unequivocal, and that we may be absolutely certain, we will endeavor to introduce this sound through the urethra into the bladder. This little operation, if carefully conducted, is generally painless. The sound enters, as you see, easily enough, until it reaches the membranous portion of the urethra, and here its course is suddenly arrested. No reasonable force will carry it any farther. Here it meets some obstruction, which feels hard and unyielding, and is sufficient to account for all the trouble this man suffers.

Here, then, we have a case of organic stricture of the urethra, of three years standing, in a patient 53 years old, and otherwise healthy. Now this condition of things has not come on suddenly. This patient has had this trouble for three years, gradually increasing, until finally, the urine escapes only in driblets. Three years ago, from some cause or other, this man suffered an inflammation of the urethra. This inflammation resulted in the effusion of coagulable lymph beneath the mucous lining of the canal, and this lymph, becoming organized, encroaches upon and nearly closes it. This form of the disease is termed organic, and is always the result of inflammation of some portion of the mucous lining of the urethra. Generally, indeed almost always, this inflammation is neither more nor less than an imperfectly cured gonorrhœa. Strictures assume different forms according to their seat, number and consistence. "In 270 specimens examined by Mr. Henry Thompson, there were 320 distinct strictures. Of these, 215, or 67 per cent. of the entire number, were situated at the junction of the membranous and spongy portions of the urethra; 51, or 16 per cent., in the centre of the spongy portion, and 54, or 17 per cent., at the external orifice and within two inches and a half behind this point."

Strictures also vary in their density, some being soft and elastic, others hard, dense and unyielding. Some encroach only moderately upon the canal, and produce very little trouble, others are so close as to be impermeable.

In the treatment of strictures we must be governed, for the most part, by the nature and character of the particular case with which we have to do. No one plan is applicable in every case. In some, gradual dilatation is preferable; in others, forcible rupture, incision, external division, or cauterization, may afford the best chances of a permanent cure.

In this patient, and indeed I think in a very large majority of the cases of permeable strictures, the treatment by gradual dilatation is altogether preferable. For this purpose, provide yourselves with a good set of steel sounds, numbering from 1 to 14, and beginning with the largest size that you can introduce without using force or causing much pain, continue their use, about every second day, until you have succeeded in dilating the canal so as to admit, readily, a No. 12 or 14 sound. This will require time, generally from three to six months, but when you once succeed in this manner, the result will be much more satisfactory both to you and to your patient. But even under these circumstances,—after you are able to introduce a No. 14 sound without any difficulty, and have succeeded in relieving every symptom of the disease—it will not be safe to entirely dismiss your patient. There is some remaining, in a fully dilated stricture, that will, in a certain proportion of cases, contract and eventually close the canal, even after a long time, unless the occasional use of the instrument is continued; and if you desire to secure a permanent result, let your patient provide himself with a large sized sound, and after you have taught him how to use it, instruct him to continue its use once or twice a month for at least a year. By doing this, you will, in nearly every case, effect a permanent cure.

We shall treat this patient by gradual dilatation, and you will have an opportunity of seeing the result.

CASE II. This, Gentlemen, is another case of inability to urinate, but of an entirely different character from the one we have just examined, and, as you will perceive, requiring entirely different treatment. This patient was admitted into the Hospital only a few days ago, and gives the following account of himself:

Aged 25, unmarried, general health good, with no predisposition to disease, either hereditary or acquired. Three weeks ago, whilst engaged in the pineries of Michigan, he was struck by a heavy log, which crushed him, until released by his fellow workmen. He was immediately carried to his boarding house, and a surgeon was summoned, who arrived thirty-six hours after the accident. During this time, he was unable to urinate; he only succeeded in passing a few drops of blood and, perhaps, a little urine. The surgeon, on arriving, at once introduced a catheter, affording complete relief.

His injuries seemed to be confined to the lower part of the back and to the perineum, and, excepting the difficulty of urinating, were not of a serious character. The doctor ordered an anodyne, and returned home. From that time to the present, he has not passed a single drop of healthy urine through his urethra. For a day or two after, a few drops of a sanguineous fluid, mixed with pus, would occasionally pass, after great exertion, attended with severe pain, but at no time after the accident was he able to empty the bladder. On the sixth day after the injury, an abscess formed over the middle of the gluteus maximus, which was opened and discharged pus mingled with urine. Through this sinus the urine has ever since continued to flow, not a drop coming in the usual way. Occasionally, this artificial opening becomes temporarily closed, and then he at once begins to suffer from the distressing effects of retention. At best, this affords a very imperfect and inconvenient outlet, almost always attended with pain; the urine dribbling away without his knowledge or control, soiling his clothing and perfuming everything about him. On attempting to introduce this sound, it readily passes down to the membranous portion of the urethra, where it meets an obstruction, which is perfectly impassable. All attempts to pass this point, with any kind of instrument, are futile and useless.

Here, then, we have a case of traumatic stricture of the urethra, perfectly impermeable, of only two weeks' standing, occurring in a man otherwise perfectly healthy, and giving rise to a urinary fistula, opening on the most prominent part of the gluteus maximus.

This is one of the most perplexing cases you will ever be called upon to treat. The condition of this patient is a most unfortunate one, and we must find some way of relief. This artificial passage and opening we must endeavor to close, and, if possible, to find a passage through the natural tract of the urethra. We cannot, as in the other case, use gradual dilatation, for it is absolutely impossible to introduce, in the present condition of the patient, even the smallest sized instrument.

In this case there has been, as in the other, inflammation of the canal, giving rise to the effusion of lymph with its consequences, but the cause of the inflammation being dissimilar, the effect is also different. In the one case, the disease had existed for three years and came on gradually, and was the result, probably, of a specific inflammation of the mucous lining of the urethra. In this case, the disease was brought on suddenly by a blow, and has existed for only three weeks.

It might be a question whether or no the urethra was not, at the time of the injury, completely ruptured; but I think the fact of his being able to pass a few drops of bloody urine, and the doctor's success in placing the catheter, prove conclusively that a complete rupture did not occur at that time, but rather a laceration, or partial rupture, sufficient to produce great difficulty in urinating, and giving rise to inflammation, the effusion of lymph and complete occlusion of the canal. This is the usual course of traumatic strictures.

In the treatment of this case, gradual dilatation, I repeat, cannot be employed, and the same may be said of internal incision, forcible rupture and cauterization. Our only resource is by external division, or, as it is more commonly called, "perineal section."

After consulting with the Hospital Staff, and obtaining the consent of the patient, we have decided to attempt to divide this stricture in the middle line of the perineum, and, if possible, to reopen the track of the urethra.

The patient being fully under the influence of ether, is placed in the same position as for lithotomy, and a large sized sound is introduced to the point of stricture. The incisions are made precisely in the mesial line of the perineum, and are carefully carried down until the point of the sound is reached and the urethra is laid open. Now comes the difficult part of the operation—the search for the other end of the urethra. In this case, you perceive, the upper wall of the urethra seems uninjured, and we have succeeded in following it with this small probe, which will serve as a director. On this probe, then, we incise the canal for about two inches of its extent, and still using the probe as a guide, we can now carry the sound into the bladder without any difficulty. Withdrawing the sound and substituting this silver catheter, which we intend to leave in the bladder for the present, the operation is completed.

We shall now remove the patient to his bed, apply water dressings to the wound, and give a full anodyne for the night. At your next visit you will see the result.

June 12th. Since our last visit this patient has progressed without a single unfavorable symptom. The urine continued to flow through the artificial opening for a few days, when it ceased, and began to pass through the urethra. He is now feeling quite well, and can urinate almost as freely and easily as ever. We have advised him to use a sound occasionally, for a long time, lest the perfect healing and subsequent contraction of the wound reproduce the stricture.

---

*Equivocal Compliments.*—A servant of an old maiden lady, a patient of Dr. Poole's, of Edinburgh, was under orders to go to the doctor every morning to report the state of her health, how she slept, etc., with strict injunctions always to add, in conformity to etiquette, "with her compliments." At length, one morning, the girl brought the following startling message: "Miss S——'s compliments, and she de'ed last nicht at aicht o'clock."

